# Multi-Functional Carbon Fibre Composites using Carbon Nanotubes as an Alternative to Polymer Sizing

**DOI:** 10.1038/srep37334

**Published:** 2016-11-23

**Authors:** T. R. Pozegic, J. V. Anguita, I. Hamerton, K. D. G. I. Jayawardena, J-S. Chen, V. Stolojan, P. Ballocchi, R. Walsh, S. R. P. Silva

**Affiliations:** 1 Advanced Technology Institute, University of Surrey, Guildford, Surrey, GU2 7XH, UK; 2 Advanced Composites Centre for Innovation and Science, Department of Aerospace Engineering, University of Bristol, Bristol, BS8 1TR, UK; 3 Bombardier, Airport Road, Belfast BT3 9DZ, Northern Ireland, UK

## Abstract

Carbon fibre reinforced polymers (CFRP) were introduced to the aerospace, automobile and civil engineering industries for their high strength and low weight. A key feature of CFRP is the polymer sizing - a coating applied to the surface of the carbon fibres to assist handling, improve the interfacial adhesion between fibre and polymer matrix and allow this matrix to wet-out the carbon fibres. In this paper, we introduce an alternative material to the polymer sizing, namely carbon nanotubes (CNTs) on the carbon fibres, which in addition imparts electrical and thermal functionality. High quality CNTs are grown at a high density as a result of a 35 nm aluminium interlayer which has previously been shown to minimise diffusion of the catalyst in the carbon fibre substrate. A CNT modified-CFRP show 300%, 450% and 230% improvements in the electrical conductivity on the ‘surface’, ‘through-thickness’ and ‘volume’ directions, respectively. Furthermore, through-thickness thermal conductivity calculations reveal a 107% increase. These improvements suggest the potential of a direct replacement for lightning strike solutions and to enhance the efficiency of current de-icing solutions employed in the aerospace industry.

The development of carbon fibre reinforced polymer (CFRP) composite materials with specific types of enhanced functionality are required for applications in engineering, and particularly in the aerospace industry. Conventional CFRP has relatively poor electrical and thermal conductivities as a consequence of the encapsulating insulating polymer matrix. In addition, CFRP is inherently non-isotropic in its properties, (specifically, mechanical, electrical and thermal conductivities). As a result, the in-plane properties of the CFRP are dominated by the high strength, stiff, electrically and thermally conductive fibres whilst the out-of-plane properties are dominated by the low strength, ductile, electrically and thermally insulating polymer matrix. Although the in-plane electrical and thermal conductivities are greater than the out-of-plane directions, they are still relatively poor and can limit the applications of the material. Subsequently, it is of particular interest to impart electrical and thermal functionalities in the in-plane and the out-of-plane directions of the carbon fibre composites.

The aerospace industry is an example of an industry that would benefit from electrical conductivity enhancements. Lightning strike protection for CFRP at present relies on metallic structures, typically in the form of metallic foils that are located on the upper surface on the CFRP laminate. These metallic structures are comparatively heavy and introduce manufacturing difficulties. In addition, the contrasting mechanical properties of the metal and the composite introduce additional stresses, weakening the structure. For these reasons, it is of interest to develop an alternative carbon-based conducting composite, enabling the removal of metals within these structures.

The poor thermal conductivities of the CFRP composites present issues for the aerospace industry when de-icing of the structures, as does any dimensional instability in space structures that utilise these components. Current solutions, such as bleeding heat from the jet engine or melting/preventing ice through electric circuits (*via* Joule heating) rely on conduction/convection mechanisms. The inherent poor thermal conductivity of CFRP renders these solutions energy/cost inefficient. Furthermore, CFRP structures are not as capable as aluminium in minimising fuel temperatures during cruising altitudes – creating the potential of inadvertently forming explosive vapours. Subsequently, to enhance the efficiency of current de-icing solutions and minimise fuel vapour formation, there is a desire to improve the thermal conductivity of the CFRP composites.

One promising area is utilising carbon nanotubes (CNTs) – hexagonal arrays of carbon atoms rolled into a seamless tube. They possess the ideal properties: high tensile strength (greater than carbon fibres^1^), high Young’s modulus^2^,^3^ and high electrical and thermal conductivities^4^, imparted from the strong sigma bonds between the in-plane carbon atoms and the *sp*^2^ hybridisation. In addition, they can be attached to, or grown on the carbon fibres (called - *fuzzy fibres*)^5^,^6^. Grown or attached, CNTs are not required to be distributed into a polymer matrix (where harmful functionalisation to the CNTs is necessary) and they do not increase the viscosity of the polymer matrix to the detriment of the processing of the composite^4^,^7^,^8^,^9^,^10^.

There is a preference in the research community to grow the CNTs as opposed to attaching them^11^, as the quality, quantity, controllability of size^12^ and alignment of the CNTs are superior. The disadvantages of growing CNTs is the reduction of the mechanical properties of the underlying carbon fibres when conventional growth techniques are used^13^. Previously, we reported a photo-thermal chemical vapour deposition (PT-CVD) growth system for CNTs on carbon fibres where only a 9.7% reduction in tensile performance was recorded^5^. However, the growth temperatures encountered in the PT-CVD system still exceeds the melting point of the polymer sizing^5^. This is a ~1 wt. % addition of a proprietary polymer (typically an epoxy of low molecular weight), applied to the surface of the carbon fibres to assist handling^14^, improve the interfacial adhesion between fibre and matrix^14^,^15^ and allow the polymer matrix to wet-out the carbon fibres^16^,^17^.

In this work, we demonstrate that CNTs provide the necessary functionality for the aerospace industry, whilst replacing the polymer sizing typically applied to carbon fibres. The examination of the physical and mechanical properties of the CNTs as a replacement for the polymer sizing are presented elsewhere^18^. To summarise, following fibre volume fraction normalisation, enhancements of: 146% in the Young’s modulus; 20% in the ultimate shear stress; 74% in shear chord modulus and 83% in the initial fracture toughness were observed^18^.

The CNTs are grown using the PT-CVD and the resulting high density and quality of CNTs has led - without a polymer sizing - to the retention of the mechanical integrity of the carbon fibre fabric and the composite fabrication capability. Furthermore, the density, quality of CNTs and length of CNTs has vastly improved the number of electrical and thermal percolation pathways, leading to significant improvements in their properties. The fabrication of the composites (fuzzy fibre and reference samples) were implemented using an industrially relevant vacuum assisted resin transfer moulding (VARTM) process. Additional samples were produced where only the uppermost plies are modified, in analogy to the metal-foil structures currently used for lightning strike protection.

Therefore, the solution presented herein, is a direct “all-carbon” replacement for the polymer sizing that in addition provides electrical and thermal functionality ultimately showing that this approach not only offers a viable alternative for current metal-foil containing CFRP, but opens up to other industries and applications.

## Results and Discussion

### CNT growth

**Figure 1:**
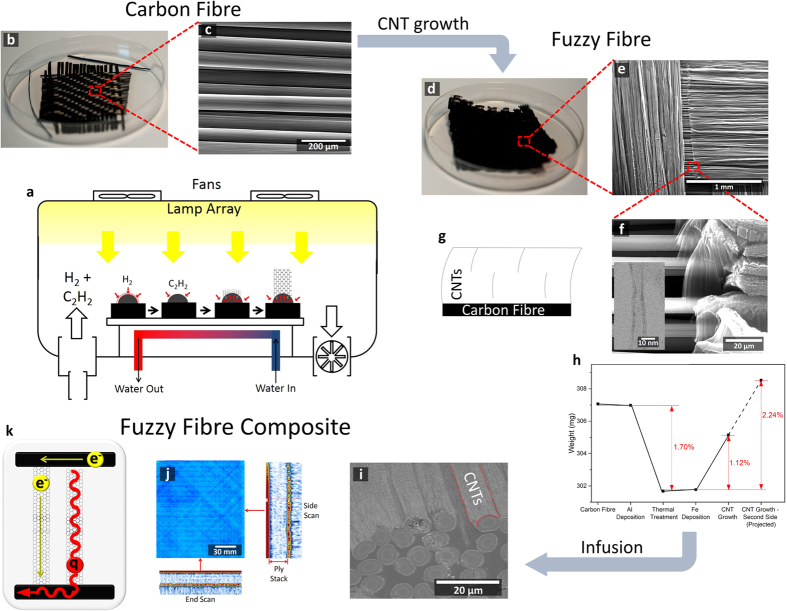
Diagram displaying the carbon fibre analysis prior and after growth of CNTs and after infusion into the final composite. (**a**) PT-CVD used for the growth of CNTs on carbon fibre. (**b**) Photograph of as-received carbon fibre with (**c**) a typical SEM image of the fibres. (**d**) Photograph of fuzzy fibre ply with (**e**,**f**) SEM images of increasing magnification. Inset of (**f**), a STEM image of a typical MWCNT observed. (**g**) Diagram displaying the location of the CNTs with respect to the carbon fibre. (**h**) Weight change during the entire CNT growth procedure. (**i**) Cross-section of the infused fuzzy fibre composite. (**j**) Non-destructive ultrasound analysis on an infused fuzzy fibre composite, blue colour signifies minimal attenuation, therefore no observable voids. (**k**) Schematic diagram describing the enhanced electron (e^−^) and phonon (q) transport from upper ply and lower plies (horizontal black lines).

The modification of the carbon fibre by the growth of CNTs is demonstrated in Fig. 1. The high density and length of CNTs is evident when comparing the photographs and scanning electron microscope (SEM) images of the as-received carbon fibre (Fig. 1b,c) and the fuzzy fibre (Fig. 1d–f) (see Supplementary Note 1 for method). Figure 1f demonstrates the highly-dense growth and aligned carbon nanotube forests structures on the carbon fibres (Fig. 1g) and is such that where growth is present, the carbon fibres are obscured. Bare carbon fibres (Fig. 1e,f) are observed, but as a result of orthogonal fibre ‘shadowing’ fibres during metal deposition. The CNTs were measured by a scanning transmission microscope (STEM) to have a diameter of 12.5 nm (inset of Fig. 1f) (see Supplementary Note 1 for method). The diameter and distinct 5-wall construction is consistent with “multi-walled” nanotube nature – the desired type of nanotube, since it features metallic conductivity. The weight change of the fabric during the growth process is displayed in Fig. 1h showing an initial reduction of weight of 1.7 wt.%, which occurs as a the result of the removal of the polymer sizing *via* a thermal annealing process^5^,^18^. The weight increase from this value is the result of weight gain by the CNT growth.

The growth of CNTs is a significant improvement over previous reports on carbon fibre, evidenced by the higher density, length and alignment of the nanotubes^5^. We report this improvement of the growth occurs partly as a result of using the Al interlayer, which is expected to minimise the diffusion of the iron catalyst in the carbon fibre substrate^19^. This interlayer also reduces the degradation to the underlying carbon fibre by the growth process^20^, acting as a thermal barrier^21^ and reducing catalyst-carbon fibre interaction, minmising pitting^11^. It is observed that the morphology of the CNTs exhibit similarities to those grown on alumina/SiC fibres^22^,^23^,^24^, which further highlights that the improvements in the CNT growth result from the Al interlayer compared to current state of the art^25^,^26^,^27^,^28^,^29^,^30^. SEM analysis of the dry fibres (Fig. 1f) and of the cross-section of the composite (Fig. 1i) (see Supplementary Note 2 for method) on the composite revealed ~40% of the fibres are covered by the CNTs (where growth is present)^18^ with CNT lengths in the range of 10–300 μm, and growth densities (where growth had occurred) of ~2 × 10^10^ tubes cm^−2^. As evident by Fig. 1i, the growth of the CNTs occurs on the outside of the collections of carbon fibres (tow). The length and alignment of the CNTs are ideal to bridge electrically and thermally insulating interlaminar regions, dominated by the polymer matrix (Fig. 1k). The infusion of the fuzzy fibres led to no observable voids as demonstrated by the ultrasound testing in Fig. 1j (see Supplementary Note 3 for method), where no high attenuation zones (voids) as indicated by the colour red, were observed. The better quality, higher density and aligned CNTs give confidence that the composite produced will be of higher quality than those produced thus far in the literature.

**Figure 2:**
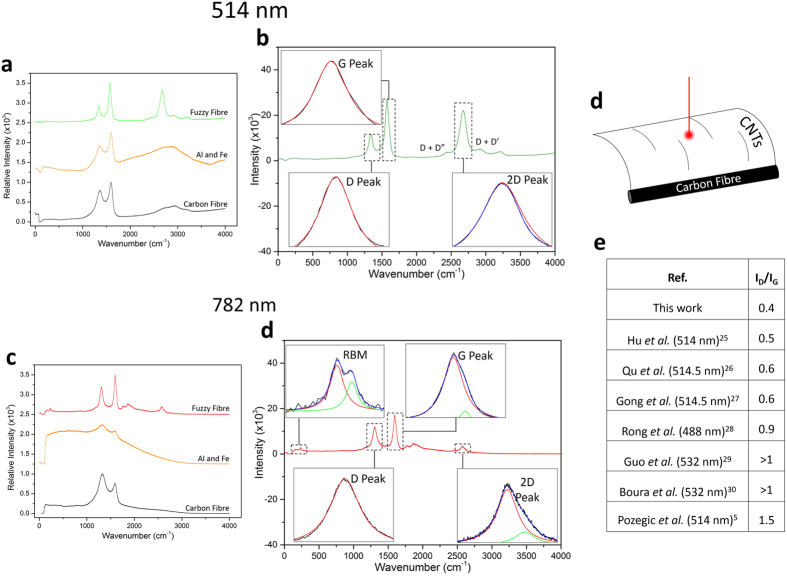
Raman spectra analysis for two laser excitation wavelengths, (**a**) 514 nm and (**c**) 782 nm and their characteristic peaks (**b**,**d**), respectively. (**a**,**c**) Raman spectra during the fabrication of the fuzzy fibres; the bare carbon fibre fabric (black curves), the metallic deposition of the aluminium interlayer and iron catalyst on the fabric (yellow curves) and the fuzzy fibre fabric ((**a**) green and (**c**) red curves). (**b**,**d**) Subsequent analysis of characteristic peaks; radial breathing mode (RBM), D, G and 2D peak for both laser excitation wavelengths. For fitted spectra, the black curves are the original spectra, green and red curves are the individual Lorentzian fits and the blue curve is the sum peak (where two curves are fitted). (**d**) The morphology of the CNTs assisted with selecting the regions to perform the spectra analysis. (**e**) Comparisons of the defect ‘D’ and the graphitisation peak ‘G’ ratio compared to other studies of CNTs on carbon fibre. Please note references (25-30, 26, 27, 28, 29, 30, 5) to comparable Raman analysis on CNT is indicated in (**e**).

The nature of the graphitic structures considered in our investigation was assessed using Raman spectroscopy (Fig. 2) with two laser excitation lines, 514 nm (Fig. 2a,b) and 782 nm (Fig. 2c,d) (see Supplementary Note 4 for method). This technique is non-destructive, and highly suited for symmetric homo-atomic lattice structures such as CNTs and carbon fibres. Fig. 2(a,c) displays the spectra for unmodified carbon fibres (black curves), carbon fibres after metal catalyst deposition (yellow curves) and for the fuzzy fibres after CNT growth, for 514 nm and 782 nm laser excitation lines (green curve and red curves, respectively).

The unmodified carbon fibre is characterised by two peaks, which are observed for both types of laser excitation energies. These peaks correspond to: a predominantly defect induced double resonance band (D- peak, 1330–1360 cm^−1 ^31) and a graphitic peak (G peak 1580 cm^−1^), albeit with different relative intensities. However, for the 514 nm laser (Fig. 2a), a broad peak is observed in the spectra of the carbon fibre at high frequencies (~2900 cm^−1^), these features are also observed for the Al and Fe modified carbon fibre. In that frequency zone, the fuzzy fibre spectra displays the 2D peak (and various combinations of overtones and intra-valley processes). The Al and Fe modified carbon fibre using the 782 nm laser (Fig. 2c) displays a broad peak which could be the result of enhanced luminescence from the metallic surface. After CNT growth, the D and G peaks featured narrower FWHM values and reduced I_D_/I_G_ ratio. This suggests the CNTs feature fewer lattice defects than the carbon fibre – with and without the metallic interlayer and catalysts. Excitation using the 782 nm laser source (Fig. 2c) displays the M and iTOLA bands, (1769 cm^−1^ and 1861 cm^−1^, respectively) for the fuzzy fibre sample. The iTOLA band is highly energy dispersive^32^ and is attributed to the combination of two intra-valley phonons – one optical mode and one acoustic mode^33^. The M band is an overtone of an infrared (IR) active mode in graphite and is a second-order process^33^. A weak, radial breathing mode (RBM) is observed in the spectrum of fuzzy fibres using the 782 nm laser source, which indicates the presence of a small number of SWCNTs^34^. This is unique to the low temperature PT-CVD process and is a signature of high quality CNT growth on any surface.

We note that the analysis of the carbon fibre surface also contained polymer sizing. In addition, the basal planes of the carbon fibre may have been altered as a consequence of the CNT growth temperature, affecting the turbostratic structure, which would be observed as a narrowing of the 2D peak. Each of the main resonant peaks observed for the fuzzy fibre samples were fitted to Lorentzian functions curve(s) in order to determine the number of peaks and their position for both excitation sources (Fig. 2b,d). The values of the peak positions are shown in the Supplementary Table S1.

The spectra obtained from the 514 nm laser excitation (Fig. 2b) shows the D and G peaks fitted with single Lorentzian curves, whilst the 2D peak was fitted to two curves. A defect ratio is obtained by the ratio of the peak intensities from the D and G peaks, (I_D_/I_G_) (Fig. 2e). The I_D_/I_G_ ratio shows that the CNTs grown in this work are of a high quality, and feature minimal structural defects, indicated by low I_D_/I_G_ values. The single fitting of the G peak suggests MWCNTs (no G^−^/G^+^ splitting) of high quality (lack of D’-peak (~1620 cm^−1^)). This multi-walled nature of the tubes corroborates with the STEM observations (inset of Fig. 1f). The 2D peak is an indicator of the stacking of the graphitic layers within the MWCNTs and can be used to determine the quality of CNTs by comparing peak intensity to I_D_ (I_D_/I_2D_). The I_D_/I_2D_ for both laser wavelengths are shown in the Supplementary Table S1. Interestingly, the single (albeit weaker) fitting of the 2D is a feature of graphene (or SWCNTs), however, it could also be expected for a few-walled thick MWCNTs of high quality stacking.

Considering the spectra data for the 782 nm laser, the feature at ~231 cm^−1^ (Fig. 2d) is assigned to a RBM. Two peaks are observed, where the second one is at 240 cm^−1^ (red and green curves). This suggests the presence of a small proportion of SWCNTs. The radii of the SWCNTs deduced from *ω*_*RBM*_ = (*A/d*_*t*_) + *B* are 1.07 nm and 1.03 nm^35^. Where *ω*_*RBM*_ is the Raman frequency shift of the RBM in wavenumber (cm^−1^) and *d*_*t*_ is the diameter of the CNT. *A* and *B* are parameters that have been empirically determined to be 234 cm^−1^ nm and 10 cm^−1^ 34, respectively, for SWCNTs with diameters between 1 and 2 nm^36^. The spectra reveal a single Lorentzian fitting for the D peak, however, the G peak in Fig. 2b shows the presence of two peaks: 1595 cm^−1^ (red) and at 1619 cm^−1^ (green). The dominant peak (1595 cm^−1^) is assigned to the G band and the small feature at 1619 cm^−1^ is assigned to the D’ peak, which resides at 1620 cm^−1^. This disorder peak was not observed for the 514 nm laser excitation energy, although it should be stated that the peak is sensitive to the area of the fibre and the chirality lattice orientation^30^. The lack of G^−^/G^+^ splitting suggests the presence of MWCNTs, as corroborated the STEM measurements. An asymmetric 2D peak of lower intensity is also observed in the fitted spectra, alluding to the possibility of weak carbon interlayer interactions.

To summarise the Raman analysis, the spectra suggest the samples are composed primarily of few-walled MWCNTs, with a small presence of SWCNTs. Although a weak 2D peak (514 nm) and a RBM spectra (782 nm) were observed (suggesting SW-), a single G peak (1575 cm^−1^ and 1595 cm^−1^ for the 514 nm and 782 nm excitations, respectively) and 2 peak fitting for the 2D peak (782 nm) were also observed (suggesting MW-). The CNTs produced in this work are of high quality (I_D_/I_G_ = 0.4), including comparisons to previous work using the same system, where broader D peaks, I_D_/I_G_ > 1 and small intensity 2D peaks were observed^5^. The motivation to incorporate CNTs in the composite is driven by the promising theoretical predictions on mechanical, electrical and thermal properties of CNTs^37^. Defective CNTs would merely incorporate defective carbon structures without the desirable properties.

Limitation of Raman spectroscopy was demonstrated by Tuinstra *et al.*^38^, who calculated that typically 70% of the Raman signal originates from the top ~250 Å and 90% from ~500 Å depth. In addition, the signal could originate from defective parts of the CNT, such as parts that are deformed by the catalyst. Fortunately, a benefit of the high density and length of the CNTs grown was the ability to target sections of the CNTs using the optical microscope and the laser (Fig. 2d). In addition, the fuzzy fibre fabric was scanned over multiple areas in order to obtain a systematic and averaged understanding of the surface which will extend the properties uniformly to composite CFRP samples too.

### Electrical Measurements

**Figure 3:**
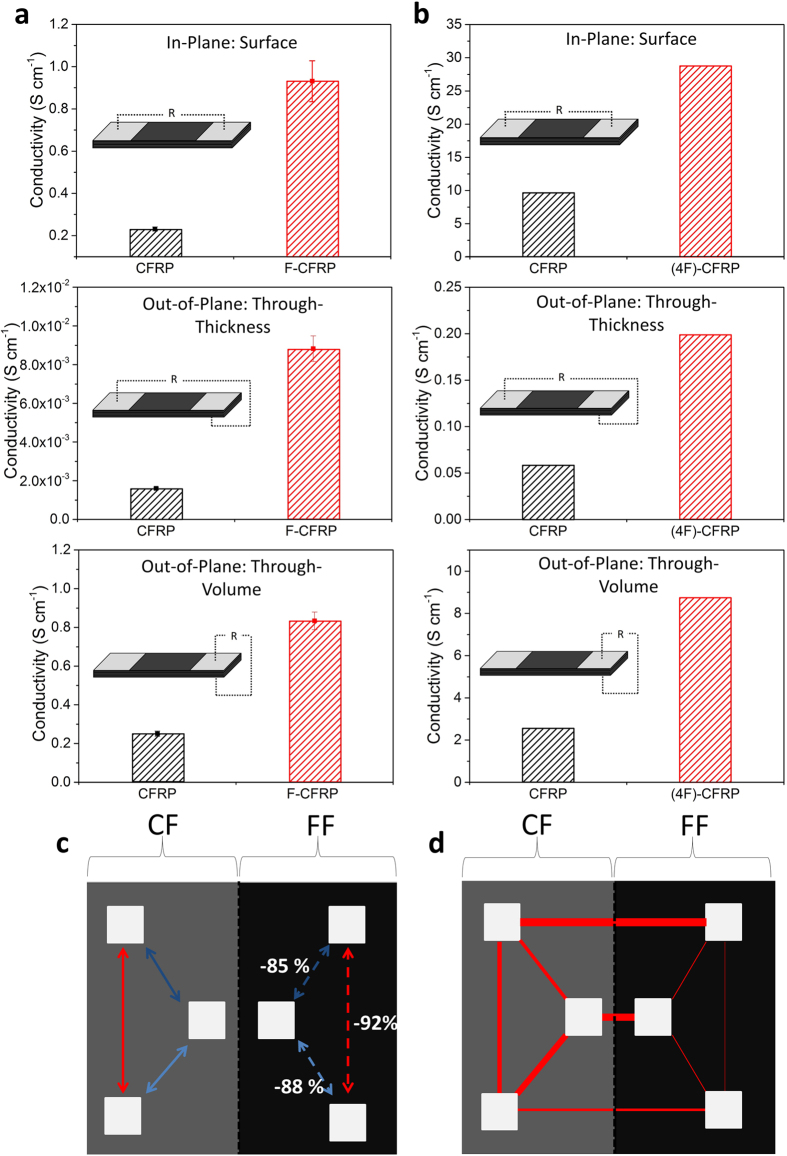
Electrical conductivity results for CFRP and F-CFRP for the surface, thickness and volume directions. (Insets of (**a**) and (**b**)) Different configurations for the electrical conductivity test. The silver rectangles on the sample depict the silver DAG conductive paint. (**a**) Electrical conductivity results for all plies modified against unmodified composite. (**b**) Electrical conductivity tests of 4-ply (of 14) FF layers (termed (4 F)-CFRP). (**c,d**) Diagram of the hybrid (carbon fibre grey, left and fuzzy fibre black, right) test specimens with electrical conductivity results. Silver squares represent the silver DAG contact patches. (**c**) Comparison between CFRP (bold lines as benchmarks) and (1F)-CFRP (dashed lines). (**d**) Comparison between all measurements where the line thickness dictates the resistance (200:1).

Electrical conductivity tests were conducted in the surface, through-thickness and volume directions and the results are displayed in Fig. 3. The figure shows strong improvements in the electrical conductivity in all directions after the addition of the CNTs. These are: 300% increase in the surface direction, 450% increase in the thickness direction and 230% increase in the volume direction for fuzzy CFRP (F-CFRP) compared to the standard CFRP. The improvement in the surface direction suggests the CNTs are bridging the insulating gap from the fibre to the electrical probe and/or utilising adjacent plies *via* CNTs to transport electrons. The greatest improvement is in the thickness direction, as the CNTs bridge the electrically insulating interlaminar regions and ply to electrical probes. This result is of key significance in developing the out-of-plane electrical properties of CFRP.

For the aerospace industry, a direct replacement of the metallic foil is achieved by modifying only the uppermost plies. Figure 3(b) shows for the 4-ply (out of a total of 14 plies) modified samples ((4F)-CFRP), significant improvements in electrical conductivity for not only the surface but, also for the thickness and volume directions. There were improvements of 200% and both 240% in the surface, thickness and volume directions for the fuzzy fibre sample, respectively. The improvements in the thickness and volume directions are a result of the migration of the CNTs to subsequent plies, or electrical percolation pathways forming within the interlaminar regions. Since the electrical contact areas are laser-ablated at external contact points (contact resistances from electrical probe to carbon fibre is negligible), the improvements in electrical conductivity in the surface direction suggests electrical pathways forming in the interlaminar regions in the uppermost plies. Additionally, improvements may also originate from the top single ply if the CNTs grown form electrical contacts on the same ply, increasing the number of electrical percolation pathways.

A hybrid (CFRP/(1 F)-CFRP) composite sample (14 plies thick) fabricated with a (50%) single fuzzy fibre plain weave and a (50%) standard plain weave ply was produced (two separate pieces, infused alongside each other). The electrical conductivity results are shown in Fig. 3c,d; the left side (grey colour) of the samples represents the unmodified CFRP, whilst the right side (black colour) represents the fuzzy fibre. The test consisted of two parts: investigating differences in conductivity across the two sections (Fig. 3c) and investigating the consistency in results (Fig. 3d). Figure 3c displays resistance (normalised by electrical probe separation) once comparing symmetrical measurements about the carbon fibre/fuzzy fibre divide (*i.e.* respective colours where bold lines are the benchmark measurements). Evidently, the single fuzzy fibre ply has led to 85–92% reductions in the electrical resistance.

Figure 3d displays the relative differences in resistance (normalised by electrical probe separation) by the thickness of the lines with (thicker the lines, the more resistive, 200:1). The inconsistency observed for the two large lateral measurements could be a result of the discontinuity of the fabric. Nonetheless, the results with just a single fuzzy fibre ply evidently demonstrate that CNTs improve the electrical conductivity in the surface direction.

Typical electrical conductivity values of standard carbon fibre composites are ~1^−1^–10^2^ S cm^−1^ in the in-plane direction and ~10^−3^–10^−2^ S cm^−1^ in the through-thickness direction^10^,^39^,^40^, which are comparable to those in reference samples in Fig. 3. The enhancements in electrical conductivity were observed for the CNT-modified carbon fibre composites for different ply lay-ups, test configurations and across different geometries.

The results of Fig. 3 suggest that the improvements in surface conductivity are the combination of CNTs bridging the insulating layer from ply to electrical probe, CNTs bridging adjacent plies, and/or increased number of intralaminar percolation pathways.

This data compares favourably with previous reports of CNT-modified carbon fibre composites. Lee *et al.*^40^ reported increases of 42% and 54% in the surface and thickness directions after depositing MWCNTs on carbon fibre *via* electrophoresis. Bekyarova *et al.*^41^ reported a maximum of 24% and 30% improvement in the surface and thickness direction respectively, after attaching MWCNTs to carbon fibre *via* electrophoresis. Enhancements in the electrical conductivity were reported by Veedu *et al.*^23^ on SiC fibres reporting improvements of 360% and 440% in the in-plane and in the through-thickness directions, respectively. Significant improvements for CNT-modified alumina fibre composites were reported by Yamamoto *et al.*^10^ of ~1 × 10^8^% in the in-plane and ~3 × 10^9^% in the out-of-plane direction.

### Thermal Conductivity Measurements

**Figure 4:**
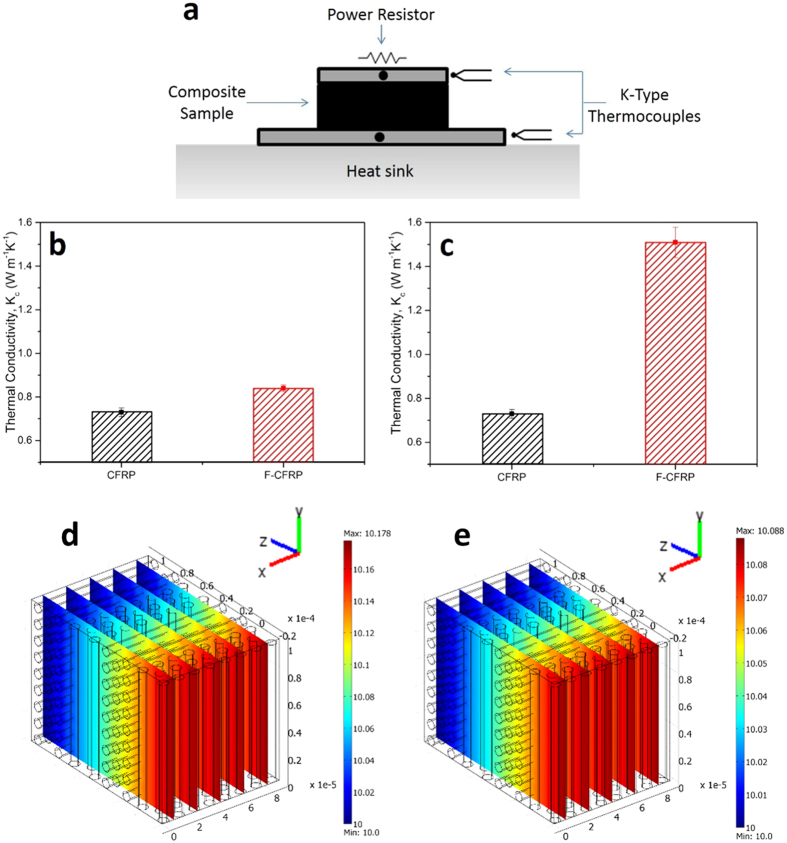
(**a**) Schematic diagram of the thermal conductivity set-up. The power resistor was placed above the top aluminium plate and thermocouples were placed in the aluminium plates above and below the sample. The aluminium disk placed at the bottom acted as a heat sink and was in contact with a water-cooled base. (**b,c**) Thermal conductivity results in the out-of-plane thickness direction for the CFRP and F-CFRP. (**b**) is the non-V_f_ normalised result and (**c**) is the V_f_-normalised. (**d,e**) Finite element thermal model of bi-axial CFRP materials in (**c**) using a heat-flow of (1 kW m^−2^) in the perpendicular direction to the fibre axes, and constant heat-sink temperature of 10 °C, for (**d**) unmodified CFRP, exhibiting a temperature rise of 0.178 °C (13.48 °C cm^−1^) and (**e**) F-CFRP, exhibiting a temperature difference of 0.088 °C (6.82 °C cm^−1^). For both models, the thermal conductivity of the fibres was 7 W m^−1^K^−1^.

CFRPs are typically characterised by poor anisotropic thermal properties. The thermal conductivity is especially lower in the through-thickness direction compared to the in-plane direction (which is dominated by the fibres), therefore thermal conductivity tests were performed on the CFRP and the F-CFRP samples in the out-of-plane direction. The power was varied (6 × 10^−2^ W to 4 W) and 10 results obtained for the CFRP and 8 for the F-CFRP, respectively. The thermal conductivity results that were extracted from the gradients of the respective graphs are presented in Fig. 4.

Prior to fibre volume fraction (*V*_*f*_) normalisation, the F-CFRP sample shows an increase in the thermal conductivity in the out-of-plane thickness direction by 15% over the standard, CFRP sample. This is a remarkable result considering the difference in *V*_*f*_ between each sample (fibre volume fraction of (48.5 ± 0.5)% for the CFRP and (20.0 ± 2.0)% for the F-CFRP used in this test^18^.

To normalise these results by the *V*_*f*_, an expression derived by Hatta *et al.*^42^ was used:


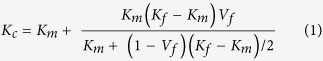


where *K*_*c*_, *K*_*m*_, *K*_*f*_ are the thermal conductivities of the composite, matrix and reinforcing fibres, respectively. This compares to using a simple expression derived from the slab model which has stated to be less reliable^43^. A value for the epoxy resin (assumed to be isotropic) of 0.3 W m.K^−1^ was obtained from Hull *et al.*^43^ and a value for *K*_*f*_ for unmodified fibres was obtained from the manufacturer to be 7 W m^−1^K^−1^ (PAN fibres are isotropic in the transverse direction^44^). The normalised results are presented in Fig. 4c. The errors were calculated by determining the distribution using the standard errors associated with the *V*_*f*_ measurements.

After normalisation, the F-CFRP displays a 107% improvement in thermal conductivity in the thickness direction. The radially grown CNTs on the carbon fibre are expected to enhance phonon coupling; reducing scatter from the interlaminar regions that is dominated by the thermally insulating polymer matrix. Yamamoto *et al.*^10^ reported a doubling in thermal conductivity in the thickness direction for their CNT coated alumina fibres using the IR microscopy method, whereas, Veedu *et al.*^23^ reported a 50% increase in thermal conductivity in the out-of-plane thickness direction for their SiC fibres. Liang *et al.* grew carbon nanofibres on carbon fibre and using the 3ω technique reported improvements of 33% in the thickness direction^45^.

CFRPs typically display poor and anisotropic thermal conductivities; normally ~10 W m^−1^K^−1^ in the in-plane and, in accordance with the reference sample in Fig. 4b,c, ~1 W m^−1^K^−1^ in the through-thickness direction^10^,^39^. MWCNTs are the ideal type of CNT as the concentric cylindrical structure reduces phonon scattering^46^,^47^. Characterisation conducted on the CNTs would suggest that they possess the ideal qualities for improving the thermal conductivity, as longer CNTs accommodate a wider range of phonon frequencies, increasing their thermal conductivity^46^. But the enhancement observed could be a lower bound improvement. The low *V*_*f*_ of the F-CFRP and the SEM images of the cross-section suggests the presence of resin rich interlaminar regions. By designing the composite so the CNTs are physically in contact will reduce phonon scattering further from the polymer matrix^48^. This feature is not accounted for in the *V*_*f*_ normalisation, suggesting that further improvements are possible. Theoretically determined improvements of ~400% for fuzzy fibre composites (CNT volume fraction of 4.27%) have been determined^49^

Finite element analysis (COMSOL Multiphysics, heat-transfer module) was used to simulate the flow of heat along the perpendicular direction to the fibre axis (positive z-axis direction in Fig. 4d,e) of the bi-axial CFRP materials used in Fig. 4c. Figure 4d shows that for the unmodified CFRP, the temperature difference required for this heat-flow is 0.178 °C across the z-dimension of the model, 130 μm. This equates to a temperature difference of 13.48 °C cm^−1^, and a thermal conductivity of 0.742 W m^−1^K^−1^ along this direction, which is in close agreement with Fig. 4c.

The thermal conductivity of the “effective matrix medium” was derived by matching the conductivity of the model to the experimentally determined value for F-CFRP (1.45 W m^−1^K^−1^ from Fig. 4c), by altering only the thermal conductivity of the matrix (the conductivity of the fibres remains constant at 7 W m^−1^K^−1^). This analysis revealed the thermal conductivity of the “effective matrix medium” to be 1.02 W m^−1^K^−1^ (Fig. 4e), which equates to a fibre-fibre conductivity enhancement factor in the out-of-plane direction of ~340%, mediated by the CNTs on the fuzzy fibres. This strong enhancement suggests that the CNTs are effectively enhancing phonon transport across adjacent fibres in the out-of-plane direction.

## Conclusions

CNTs are shown to be a suitable replacement for the polymer sizing ubiquitous on fibres with the additionality of also providing the potential of enhanced electrical and thermal functionality for industries that are compromised with metallic structures. This has been due to high quality, densely grown CNTs which were, in part, a result of the aluminium interlayer which has previously been shown to minimise the diffusion of the iron catalyst in the carbon fibre substrate, thus retaining the activity of the catalyst.

The F-CFRP displayed strong improvements in electrical conductivity in all three directions; with significant enhancements in the surface (300%), thickness (450%) and volume (230%) directions. The improvement in the surface direction is significant and suggests the formation of additional intra- and interlaminar electrical percolation pathways, providing many new routes and multiple applications. The thermal conductivity was observed to improve by 107% in the thickness direction after fibre normalisation and display an improvement of 15% prior to normalisation. This is remarkable given the *V*_*f*_ differences; the F-CFRP displays a 15% improvement for a composite with 40% of the V_f_ of the CFRP. The CNTs exhibit high quality when compared to other reports, allowing efficient electron and phonon transport with the carbon fibre in the out-of-plane directions through otherwise thermally and electrically insulating epoxy resin domains. We therefore suggest the fuzzy fibres methodology presented offers a suitable alternative to the polymer sizing and in doing so, potentially a first step in removing the reliance on metallic structures in aircrafts.

For future work, the scalability of this technology should be considered. CNTs have been grown on a moving substrate without affecting the growth of CNTs, suggesting the possibility of a roll-to-roll fabrication of fuzzy fibres in the future^50^. By growing the CNTs during the carbon fibre fabrication or the surface treatment stage, it could potentially further minimise damage to the carbon fibres.

## Methods

### Fuzzy Fibre Fabrication

For the fabrication of the fuzzy fibres, 35 nm of Al was deposited using a DC magnetron sputtering system (JLS MPS 5000) on both sides of the carbon fibre fabric to be thermally treated at 800 °C under a flow of H_2_ (100 sccm) for 15 minutes. The catalyst for CNT growth was iron (6 nm), deposited using DC magnetron sputtering on one side of the carbon fibre fabric prior to CNT growth on the same side.

CNT growth on the carbon fibre (Grafil Inc. Pyrofil TR30s 2/2 twill) was performed using a PT-CVD (Surrey NanoSystems 1000n) system (Fig. 1a). The system features a CVD chamber with a water-cooled substrate table and walls (held at 10 °C *via* a closed-loop water cooling system) and top-down optical heating (maximum power of 8 kW)^51^. The water-cooled substrate table reduces the temperature of the carbon fibre cloth whilst only the top layer of the fibre is exposed to the top-down heating, necessary to enable high quality CNT growth^52^. In addition, hydrogen was introduced during the entire growth process to provide convection cooling. This system has been used previously for this purpose, and has already demonstrated minimal thermal degradation of the carbon fibre from the CNT growth process^5^. This contrasts with standard thermal CNT growth systems, where a significant reduction in carbon fibre tensile strength has been observed^15^,^16^,^53^,^54^. The growth procedure consists of a reduction for the iron oxide using hydrogen gas at 750 °C and pressures of 10 Torr as acetylene gas was used as the carbon feedstock and introduced 10 minutes into the procedure. Temperature measurements obtained using a thermocouple in contact with the top-surface of the carbon fibre typically measured 800 °C during the CNT growth. As well as reducing the temperature of the carbon fibre, the cooling mechanism reduces the process time between samples, increasing sample through-put. Once growth was completed, iron (6 nm) was deposited on the reverse side of the carbon fibre fabric and the growth process repeated.

A vacuum-assisted resin transfer moulding (VARTM) process was used to infuse the fabric (unless otherwise stated) with the polymer matrix (see Supplementary Figure S1). For this procedure, a flat metal mould was initially treated with releasing agent. The carbon fibre ply stack was subsequently positioned on the metal mould between two peel plies - above and beneath the stack. The peel plies were sandwiched between two infusion meshes (above and beneath the peel plies). Two silicone connectors were placed on the metal mould with the stack placed between them and a spiral feed placed along the leading edge next to an epoxy resin container. The entire stack, silicone connectors and spiral feed were encapsulated by vacuum bagging and adhered to the mould with vacuum bagging tape. Two PVC pipes were connected to the silicone connectors; one to a vacuum pump *via* a resin catchpot and the other pipe to the epoxy resin container. The epoxy resin was a low viscosity bisphenol-based epoxy resin, (‘IN2 Epoxy Infusion Resin’ (EasyComposites)).

### Determination of Fibre Volume Fraction

The fibre volume fraction (*V*_f_), was determined using thermal gravimetric analysis (TGA)^55^ (see Supplementary Note 4). Preliminary testing was performed to determine fibre stability and polymer matrix decomposition for the TGA process. The weight (%) in correlation to time of the TGA process for each material (and epoxy resin) can be seen in the Supplementary Figure S2. In all cases, the final temperature was 600 °C under a nitrogen atmosphere. Measurements were performed on unmodified and modified composite types and samples with different fabric lay-up. Both standard CFRP (n = 3) and modified fuzzy CFRP (F-CFRP) (n = 6) CFRP were tested, using the bulk epoxy resin (n = 2) to determine the residue mass.

### Electrical Conductivity Measurements

Composite samples with the lay-up [0/90]_4_ were cut into to the dimensions of (40 × 20) mm and electrical contacts added using conductive paint (silver DAG), producing an electrode area of (10 × 20) mm and enabled in- and out-of-plane conductivity measurements. Samples were abraded with silicon carbide paper on the edges and cleaned with isopropanol to remove any traces of the conductive paint from shortening the contacts in the thickness and volume directions. Current-voltage (I-V) measurements were obtained from a Keithley 4200-SCS with tungsten tips using a two-probe measurement configuration.

In addition, further electrical conductivity tests were performed on samples fabricated using a proprietary resin transfer infusion (RTI) process by Bombardier. Four and a single plain-weave fuzzy fibre plies (140 × 140) mm were stacked with unmodified carbon fibre plies in the following configurations: [FF/FF/FF/FF/±45/0/0/90/0/0/90/±45/0/0/±45/0/90] and [FF/±45/0/0/±45/90/0/0/90/±45/0/0/±45/0/90], where FF refers to fuzzy fibres. Once infused the composites were cut into (75 × 50) mm sized samples, and an area of (50 × 12.5) mm was laser ablated on the ends, top and bottom to remove the surface resin and expose the topmost carbon fibre. The laser ablation was required to produce a suitable electrical contact with the fibres and furthermore, replicates the resin burn-off at the surface of the CFRP that takes place during a lightning strike. The conductive epoxy resin was subsequently used to adhere the aluminium tabs on the laser-ablated areas. A four-point probe system was used to determine the resistance of the material (Resistomat 2318).

### Thermal Conductivity

A steady-state experimental method was used to measure the thermal conductivity of the samples ([0/90]_16_) in the through-thickness (out-of-plane) direction for CFRP and the F-CFRP (all plies modified). The CFRP sample was sandwiched between two 2 mm-thick aluminium plates, using a thin layer of silver epoxy for bonding. Thermal energy was supplied to the top Al plate using a power resistor (1.5 ± 5% Ω BPC10 resistor, rated at 10 W), with the bottom aluminium plate connected to an aluminium heat sink. The ensemble was placed in vacuum to minimise heat transfer by convection. The thermal conductivity was determined from: the temperature difference between the aluminium plates (measured using K type thermocouples) at thermal equilibrium, the power supplied to the resistor, and the thickness of the sample.

A range of power inputs were used in order to generate different temperature changes between the upper and lower thermocouple. The thermal conductivity was calculated using *k=(Q·x/ΔT·A*), where *k* is the thermal conductivity, *Q* is the power input, *x* the thickness of the sample and *A* is the cross-sectional area of the sample.

### Finite Element Analysis of Thermal Conductivity

COMSOL Multiphysics (heat-transfer module) was used to simulate the flow of heat along the perpendicular direction to the fibre axis. For the models, a heat flow-rate of 1 kW m^−2^ was injected along this direction, using a constant heat-sink temperature of 10 °C at the cold-face. Isotropic values for the thermal conductivity of 7 W m^−1^K^−1^ and 0.3 W m^−1^K^−1^ were used for the fibres and for the neat-resin matrix respectively

The finite element model of the fuzzy fibres is highly complex computationally. For this reason, it was performed by approximating the CNT-containing matrix to an “effective matrix medium” that features a different thermal conductivity to that of the neat-resin matrix in the unmodified CFRP. This approximation allows the same physical model design as for the unmodified CFRP material.

## Additional Information

**How to cite this article**: Pozegic, T. R. *et al.* Multi-Functional Carbon Fibre Composites using Carbon Nanotubes as an Alternative to Polymer Sizing. *Sci. Rep.* **6**, 37334; doi: 10.1038/srep37334 (2016).

**Publisher's note:** Springer Nature remains neutral with regard to jurisdictional claims in published maps and institutional affiliations.

## Supplementary Material

Supplementary Information
