# Supplementary material for: Multi-Functional Carbon Fibre Composites using Carbon Nanotubes as an Alternative to Polymer Sizing
Source: Sci Rep. 2016 Nov 23;6:37334. doi: 10.1038/srep37334 (PMC5120359; doi:10.1038/srep37334)
Supplement: Supplementary Information [file srep37334-s1.doc]

**Multi-Functional Carbon Fibre Composites using Carbon Nanotubes as an Alternative to Polymer Sizing**

*Thomas R. Pozegic, José V. Anguita, Ian Hamerton, Imalka Jayawardena, Jeng-Shiung Chen, Vlad Stolojan, Paolo Ballocchi, Robert Walsh and S. Ravi P. Silva*[[1]](#footnote-2).*

T. R. Pozegic^1^, J. V. Anguita^1^, I. Hamerton^2^, K. D. G. I. Jayawardena^1^, J-S. Chen^1^, V. Stolojan^1^, P. Ballocchi^3^, R. Walsh^3^ and S. R. P. Silva*^1^.

^1^ Advanced Technology Institute, University of Surrey, Guildford, Surrey, GU2 7XH, U.K.

^2^ Advanced Composites Centre for Innovation and Science, Department of Aerospace Engineering, University of Bristol, Bristol, BS8 1TR, U.K.

^3^ Bombardier, Airport Road, Belfast BT3 9DZ, Northern Ireland, U.K.

**Supplementary Notes**

**Supplementary Note 1 | Electron Microscopy**

The fuzzy fibre and unmodified carbon fibre plies were analysed by scanning electron microscopy (SEM, Quanta 200 from FEI) in fabric form with masking tape adhered around the edges to aid handling and prevent airborne fibres, which could potentially enter the vacuum pumps. SEM was performed using an FEI Quanta 200 and was operated under high vacuum with a secondary electron detector employed. Typical voltages were between 2 - 20 kV, spot sizes of 2 - 4 nm with a working distance of 10 mm.

For STEM analysis, a fuzzy fibres were placed in a vial containing isopropyl alcohol (5 ml) and ultrasonicated for 10 minutes to be subsequently drop casted on a carbon grid for imaging.

**Supplementary Note 2 | Cross-sectional Analysis**

Once the polishing process (Supplementary Table 1) was completed on the 4-ply thick fuzzy fibre composite (F-CFRP), a 7.5 nm gold film was deposited using a DC magnetron sputtering system prior to SEM analysis.

**Supplementary Note 3 | Ultrasound Testing**

Ultrasound testing was performed using an Olympus Omniscan phased array ultrasonic testing instrument with a MX 5 MHz probe in a water filled immersion tank. The sample tested was a single fuzzy fibre ply consolidated into a composite with a stack of unmodified carbon fibre plies.

**Supplementary Note 4 | Raman Spectroscopy**

Raman spectroscopy on unmodified carbon fibre ply, interlayer and catalyst-deposited carbon fibre ply, and fuzzy fibre ply samples were performed using the 514 nm and 782 nm argon-ion laser lines, operated at a laser power of 17 mW, and using a Renishaw Raman spectroscope with a Leica DM LM microscope. Scans were between 0 and 3000 cm^-1^ with a duration of 30 s and repeated twice (3 iterations). A microscope with a magnification lens of 50x, provided a spatial resolution of ~5 µm and data analysed as discussed in the literature ^1^.

**Supplementary Note 4 | Determination of Fibre Volume Fraction *via* Thermogravimetric Analysis**

The method employed was a modified version by Yee *et al.*^2^, where a variable temperature rate (see inset of Supplementary Figure 2), was employed under a nitrogen flow (60 ml/min). Initially, poly matrix samples were tested to confirm decomposition where any remaining mass was attributed to residue and refined the fibre volume fraction calculation. In addition, the carbon fibres were tested to confirm stability.

The weight percentage of the fibre (*f_f_*) was modified to account for residue after the decomposition of the polymer matrix^2^:

|  | 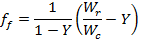 | 1 |
| --- | --- | --- |

Where *W_r_* is the weight of the polymer matrix, *W_c_* is the weight of the composite and *Y* is the percentage residue from thermally decomposing the pure polymer matrix. The fibre volume fraction (*V_f_*) was calculated using:

|  | 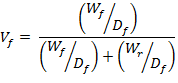 | 2 |
| --- | --- | --- |

Where *W_f_* is the weight of the carbon fibre; *W_r_* is the weight of the polymer matrix, *D_f_* is the density of the fibre and *D_r_* is the density of the polymer matrix.

For the F-CFRP, the CNTs remain on the carbon fibres, hence are included in the fibre volume fraction:

|  | 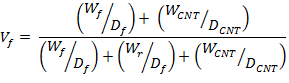 | 3 |
| --- | --- | --- |

Where *W_CNT_* is the weight of the CNTs (see Figure 1a in article) and *D_CNT_*  is the density of the CNTs (1.74 g/cm^3^ ^3^).

**Supplementary Figures**


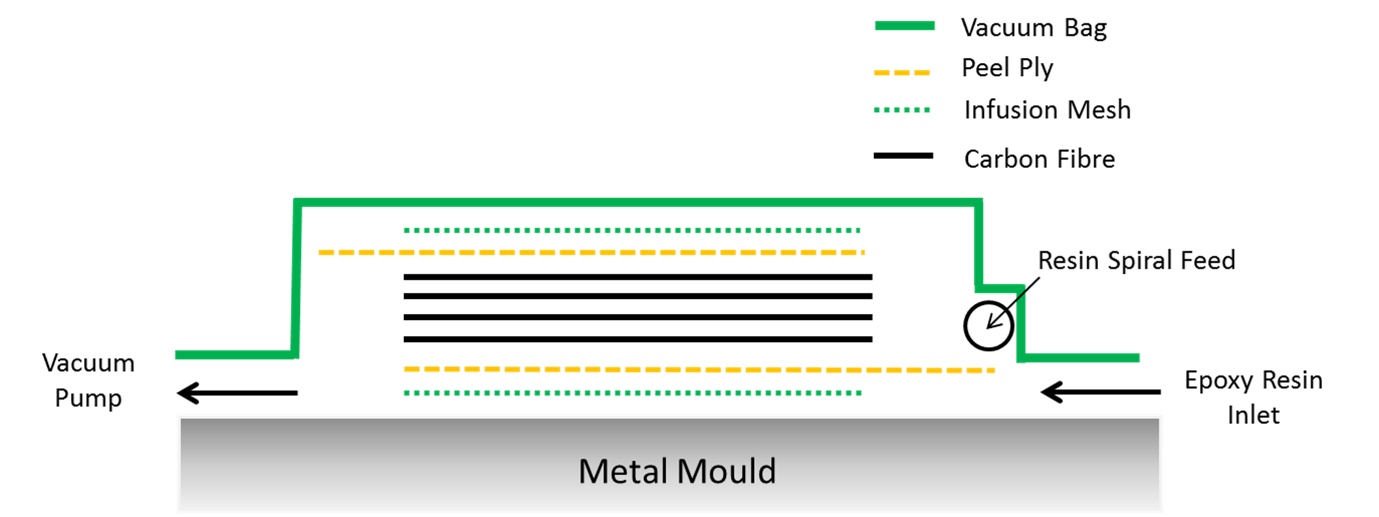


**Supplementary Figure 1 |** Schematic diagram of the vacuum assisted resin transfer moulding (VARTM).


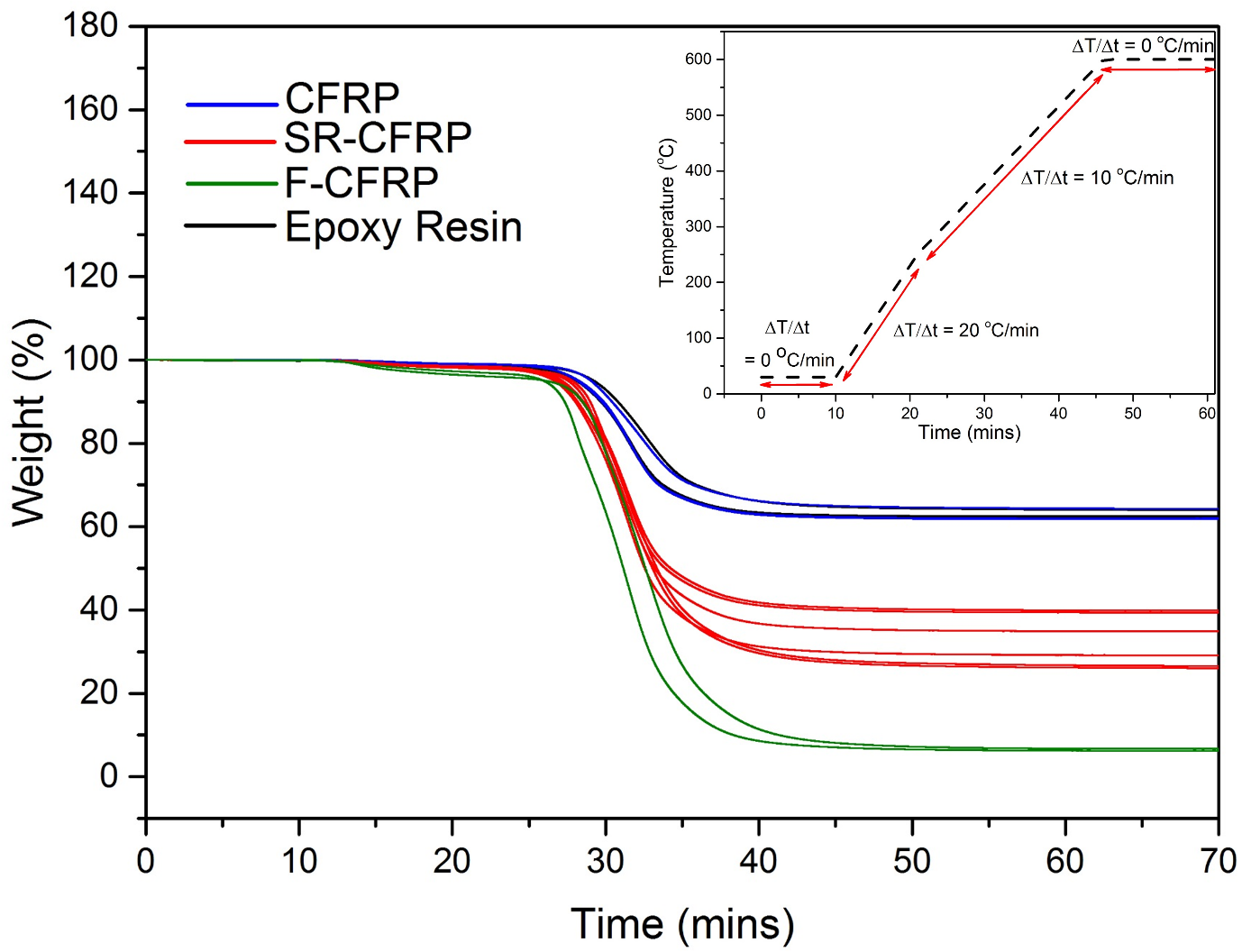


**Supplementary Figure 2 |** TGA data of weight % *versus* time to determine the fibre volume fraction of each composite. Percentages shown include residue weight. (Inset) TGA process used, carried out under a nitrogen flow (60 ml min^-1^)

**Supplementary Tables**

***Supplementary Table 1 | Key peak positions and intensity ratios to characterise the CNTs on the carbon fibre***

|  | ***Stoke Shift (cm^-1^)*** | | | | ***Intensity Ratios*** | |
| --- | --- | --- | --- | --- | --- | --- |
|  | ***RBM*** | ***D*** | ***G*** | ***2D*** | ***I_D_/I_G_*** | ***I_D_/I_2D_*** |
| ***514 nm*** | - | 1339 | 1575 | 2676, 2740 | 0.4 | 0.5 |
| ***782 nm*** | 231, 240 | 1308 | 1595 | 2575, 2619 | 0.7 | 3.5 |

**Supplementary Table 2 |** Polishing process for cross-sectional analysis.

| ***Polishing Stage*** | ***Consumable*** | ***Lubricants (Flow Rate, ml s^-1^)*** | ***Polishing Speed (rpm)*** | ***Polishing Time (min)*** |
| --- | --- | --- | --- | --- |
| 1 | Struers A/S, SiC, 200 mm, 500 Grit | Water (13) | 50 | 3 |
| 2 | Struers A/S, SiC, 200 mm, 1200 Grit | Water (13) | 50 | 3 |
| 3 | Struers A/S, SiC, 200 mm, 2400 Grit | Water (13) | 50 | 3 |
| 4 | Buehler FiberMett, 200 mm, 3 µm | Water (1) | 50 | 5 |
| 5 | Buehler, 200 mm, 1 µm, | Water (1) | 50 | 5 |
| 6 | Buehler Ultra-Prep, 200 mm, 0.5 µm | Water (1) | 50 | 5 |
| 7 | Buehler, 200 mm, 0.3 µm | Water (1) | 50 | 5 |
| 8 | Buehler FiberMet, 200 mm 0.05 µm, | Water (1) | 50 | 5 |

**Supplementary References**

1. Chen J-S, Stolojan V, Silva SRP. Towards type-selective carbon nanotube growth at low substrate temperature via photo-thermal chemical vapour deposition. *Carbon* **84**, 409-418 (2015).

2. Yee RY, Stephens TS. A TGA technique for determining graphite fiber content in epoxy composites. *Thermochimica Acta* **272**, 191-199 (1996).

3. Kim SH, Mulholland GW, Zachariah MR. Density measurement of size selected multiwalled carbon nanotubes by mobility-mass characterization. *Carbon* **47**, 1297-1302 (2009).

1. *? Corresponding author. Tel: 01483 68 9825. E-mail: s.silva@surrey.ac.uk [↑](#footnote-ref-2)
